# Rapid analysis of terpenes produced by fermentation using flow injection analysis coupled to APCI MS

**DOI:** 10.1039/d5ay02027a

**Published:** 2026-02-12

**Authors:** Bridget E. Murray, Moritz Pott, Jules Beekwilder, Robert T. Kennedy

**Affiliations:** a Department of Chemistry, University of Michigan, Ann Arbor, MI 48109, USA.; b BASF SE, Ludwigshafen Am Rhein 67056, Germany; c Isobionics BV, 6167 RD, Geleen, The Netherlands; d Department of Pharmacology, University of Michigan, Ann Arbor, MI 48109, USA

## Abstract

Terpenes are an important class of chemicals widely used in consumer and industrial processes. Enzyme- and microorganism-based synthesis is of interest for producing terpenes because of the high selectivity and relatively low environmental impact of these methods compared to other synthetic strategies. Realizing these advantages requires engineering enzymes and microorganisms for good yield of desired products. Such engineering often requires screening hundreds to thousands of genetic variants. High-throughput, label-free screening methods that provide relative quantification and can be readily adapted to new analytes are attractive for decreasing the time required for engineering. We developed a flow injection analysis (FIA) method using atmospheric pressure chemical ionization (APCI) coupled to a quadrupole time-of-flight (Q-TOF) mass spectrometer for detection of terpenes in fermentation mixtures. The method was tested by screening 99 strain variants producing a sesquiterpenoid at 1.3 injections per min with no observable carryover. Comparison of measured concentrations determined by gas chromatography-flame ionization detection (GC-FID) revealed good agreement between the methods with a correlation coefficient of 0.96. However, the GC method required 7 min per injection. Experimental parameters that can be adjusted to improve throughput for the FIA-APCI-MS are also identified.

## 1. Introduction

Terpenes are a class of nonpolar, volatile organic compounds that are relevant to the food, fragrance, and skincare industries.^1,2^ Microorganism-based terpene synthesis has emerged as an attractive production method due to its efficiency and low environmental impact.^1,3-6^ To expand its utility, there have been a number of efforts aimed at engineering more efficient terpene production by microorganisms.^7-10^ Engineering often requires the screening of hundreds^11^ to thousands^12^ of genetic variants, as it is typically difficult to know the effect of an amino acid substitution a priori.^13^ For terpenes, screening can be time-intensive if standard assays, such as gas-chromatography coupled to mass spectrometry (GC-MS),^14^ are used. Even with a GC-MS throughput of 4.6 min per run^15^ (run times of 10–40 min are more typical^16-20^ for detection of multiple compounds), it would take 19 hours to screen a library of 100 variants. This limitation has created interest in developing high-throughput screening techniques for terpenes produced by enzymes and microorganisms.^12,15,21-25^

Flow injection analysis (FIA) has been used as a method to introduce samples directly to a detector, by-passing a timeconsuming separation step.^26^ FIA can be easily automated and has been used for high-throughput detection of pharmaceuticals, pesticides, and environmental contaminants.^26^ A variety of detectors including electrochemical,^27^ UV-vis absorbance,^28^ fluorescence,^29^ and MS^30^ have been used. Of these methods, MS provides the highest information content and is most suited for terpene measurements.

Direct injection to a mass spectrometer *via* electrospray ionization (ESI) has been used for assays targeting a variety of compounds including lipids, metabolites, and antibodies.^31-34^ For nonpolar compounds which do not ionize well with ESI, alternate ionization methods have been used.^14,35,36^ FIA coupled to atmospheric pressure chemical ionization-tandem mass spectrometry (FIA-APCI-MS/MS) was used to quantify ergosterol from plasma membrane extract with 1 min analysis times and good reproducibility (RSD = 11%).^35^ Headspace injections were used to analyze terpenes produced in fermentation mixtures using proton transfer reaction-mass spectrometry (PTR-MS) at ~1 sample per min covering 4 terpene synthase variants.^14^ Other techniques may also be suitable for terpene screens. Laser diode thermal desorption with APCI has been used to rapidly analyze nonpolar compounds.^37,38^ Using this technique, a 384-well plate was analyzed in 13.7 min from 100 nL of acoustically deposited sample (30 samples per min throughput).^38^ While the analysis is high-throughput, it does require use of a consumable stainless steel well plate ($100) and samples need to be dried down prior to analysis.

In this work, we examine FIA coupled to APCI-MS for rapid (1 injection/min throughput) relative quantification of bisabolol and nerolidol (see Fig. S1 for structures) from low volume (<10 mL) fermentation extracts. Bisabolol is a valued skincare ingredient^39^ and nerolidol is used as a flavoring agent,^40^ illustrating the method's industrial utility. This method is demonstrated to be suitable for screening for high activity strain variants with relative quantification accuracy comparable to GC-FID.

## 2. Materials and methods.

### 2.1 Chemicals and reagents

All chemicals were purchased from Sigma-Aldrich (Saint Louis, MO) unless otherwise noted. 200-proof ethanol was purchased from Decon laboratories (King of Prussia, PA). All stock terpene solutions were prepared in dodecane.

### 2.2 Fermentation

Authentic fermentation samples of the sesquiterpenes were supplied by BASF and were produced as described previously.^6^

### 2.3 GC-FID of bisabolol and nerolidol

The quantification of terpene in dodecane was carried out using an Agilent 7890 GC, equipped with an Agilent DB-5 column (10 m × 0.1 mm × 0.1 μm) and a flame ionization detector. Hydrogen (99.999% purity, AirLiquide) was used as a carrier gas with a flow rate of 0.3 mL min^−1^ and the injection volume was set to 1 μL with a split ratio of 1 : 100. Initial set temperature of the column oven was 120 °C and was then heated from 120 to 240 °C at a rate of 40 °C min^−1^. Terpene quantification was performed using a standard curve of authentic standards (Merck).

### 2.4 Flow injection analysis-APCI-MS/MS

A manually operated Rheodyne six-port valve equipped with a 1 μL sample loop was used to inject samples. The sample loop was overfilled by injecting 5 μL of sample with a 25 μL Hamilton gastight syringe (Reno, NV) to ensure reproducible injections. The syringe was rinsed with dodecane between injections. For large scale experiments, samples contained in vials were arranged in order of injection prior to the start of screening. During screening, sample vial lids were unscrewed, 5 μL of sample was manually drawn up into the syringe, and sample was injected by inserting the syringe into the valve and depressing the plunger followed by flipping the valve. For most experiments, an Accela high pressure LC pump (Thermo Scientific, Waltham, MA) was used to drive flow at 1 mL min^−1^ to the ionization source. For some initial experiments, a syringe pump (Chemyx Fusion 400) fitted with a 10 mL Hamilton gastight syringe (Reno, NV) was used for flow.

Linalool in dodecane was added as an internal standard (IS) to standards and fermentation extracts so that the final linalool concentration was 100 μg mL^−1^ in samples. After addition of the IS, fermentation extracts were either left undiluted or diluted in dodecane with the IS depending on terpene concentration. For analysis, terpene concentration in fermentation extract was determined with GC-FID and known prior to FIA-APCI-MS/MS analysis. For bisabolol analysis, standards were prepared over the following concentrations in the sample matrix (1.25% dodecane in ethanol): 0, 10, 25, 75, 100, 150, 200, and 250 μg mL^−1^. For nerolidol analysis, standards were prepared at the following concentrations in the sample matrix (dodecane): 0, 5, 50, 100, 150, and 250 μg mL^−1^. All calibration standards and samples were analyzed in triplicate.

Mass spectrometric analysis was performed with a waters Xevo Q-TOF and the waters electrospray chemical ionization platform, using an ESI probe operated in APCI mode and a corona pin. The following source parameters were used: operated in current mode, corona was 5 μA, sampling cone was 15, extraction cone was 2.0, source temperature was 100 °C, probe temperature was 500 °C, cone gas flow was 130 L h^−1^, and desolvation gas was 1200 L h^−1^. MS/MS settings for valencene, bisabolol, linalool, and nerolidol are listed in Table 1.

### 2.5 FIA-APCI-MS data analysis

Signals were quantified by peak area. Peak width was measured by measuring the width at 10% of the peak height. Peak integration was performed automatically using MassLynx software. All peaks were visually inspected to ensure proper integration. Error bars shown are standard deviation of 3 technical replicates unless otherwise noted.

## 3. Results and discussion

### 3.1 Detection of terpenes with APCI-MS, APCI-MS/MS, and throughput considerations

**Fig. 1:**
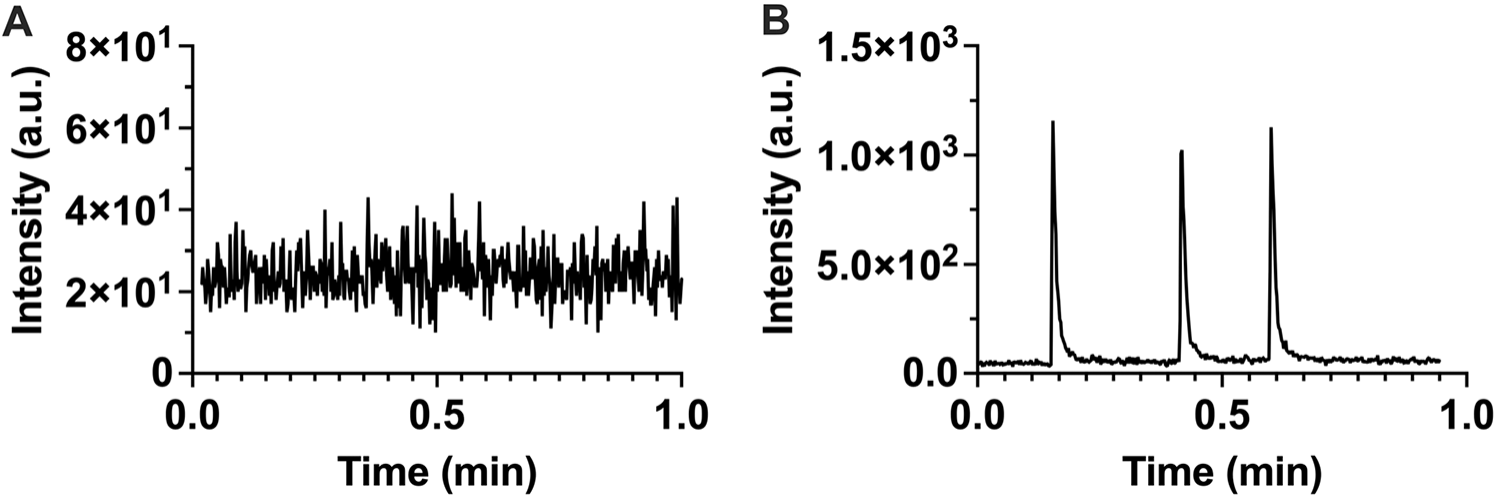
FIA-APCI-MS traces of 50 μg ml^−1^ nerolidol injected in triplicate using either (A) ESI or (B) APCI as ionization method. Traces were extracted at *m/z* = 205.2.

Fig. 1 compares FIA-MS/MS signals for 50 μg mL^−1^ nerolidol in ethanol using ESI and APCI on the same mass spectrometer. The lack of signal with ESI highlights the challenges of using ESI for terpene analysis and motivates the use of APCI for such compounds. Therefore, we pursued developing a FIA-APCI-MS/MS method with potentially higher throughput than GC-FID and tested it against samples containing terpenes produced via fermentation.

We examined experimental parameters that limited throughput of FIA-APCI-MS/MS using valencene as a test analyte for initial method development. Pilot experiments showed that the limiting factor for throughput by FIA-APCI-MS was the width of the resulting terpene peak *i.e.*, not valve loading or actuation time. Parameters such as carrier phase composition and carrier phase flow rate were found to affect peak width, while sample loop volume did not. Ethanol, as the carrier phase, led to a narrower peak width than water (Fig. S2), increasing carrier phase flow rate was found to decrease analyte peak width (Fig. S3), while the same peak width was observed between a 0.3 and 1 μL sample loop (Fig. S4). We did not investigate the reason why using water as carrier phase gives a higher and broader peak; however, we speculate the reduced dispersion of the terpene into the aqueous carrier phase due to its poor water solubility may increase signal and differences in viscosity and effects of mixing the solvents (samples were in ethanol) may contribute to the width. Increasing carrier phase flow rate to decrease analyte peak width has previously been identifled as a strategy to increase FIA method throughput.^41^ We also observe a smaller peak height when increasing flow rate (Fig. S3A and B), which is attributed to poor analyte ionization due to insufflcient liquid vaporization. As such, probe temperature was increased from 400 °C to its maximum value of 500 °C in screening experiments. Based on these results, we used ethanol as the carrier phase infused at 1 mL min^−1^ for further experiments. We also observed that analyte concentration affected peak width. When bisabolol concentration was increased 10-fold (from 25 to 250 mg mL^−1^), analyte peak width increased 2.8-fold (from 0.025 ± 0.005 min to 0.07 ± 0.01 min) (Fig. S5). The above conditions were then applied to bisabolol and nerolidol, so that the method could be tested against strain variants producing those terpenes.

**Fig. 2:**
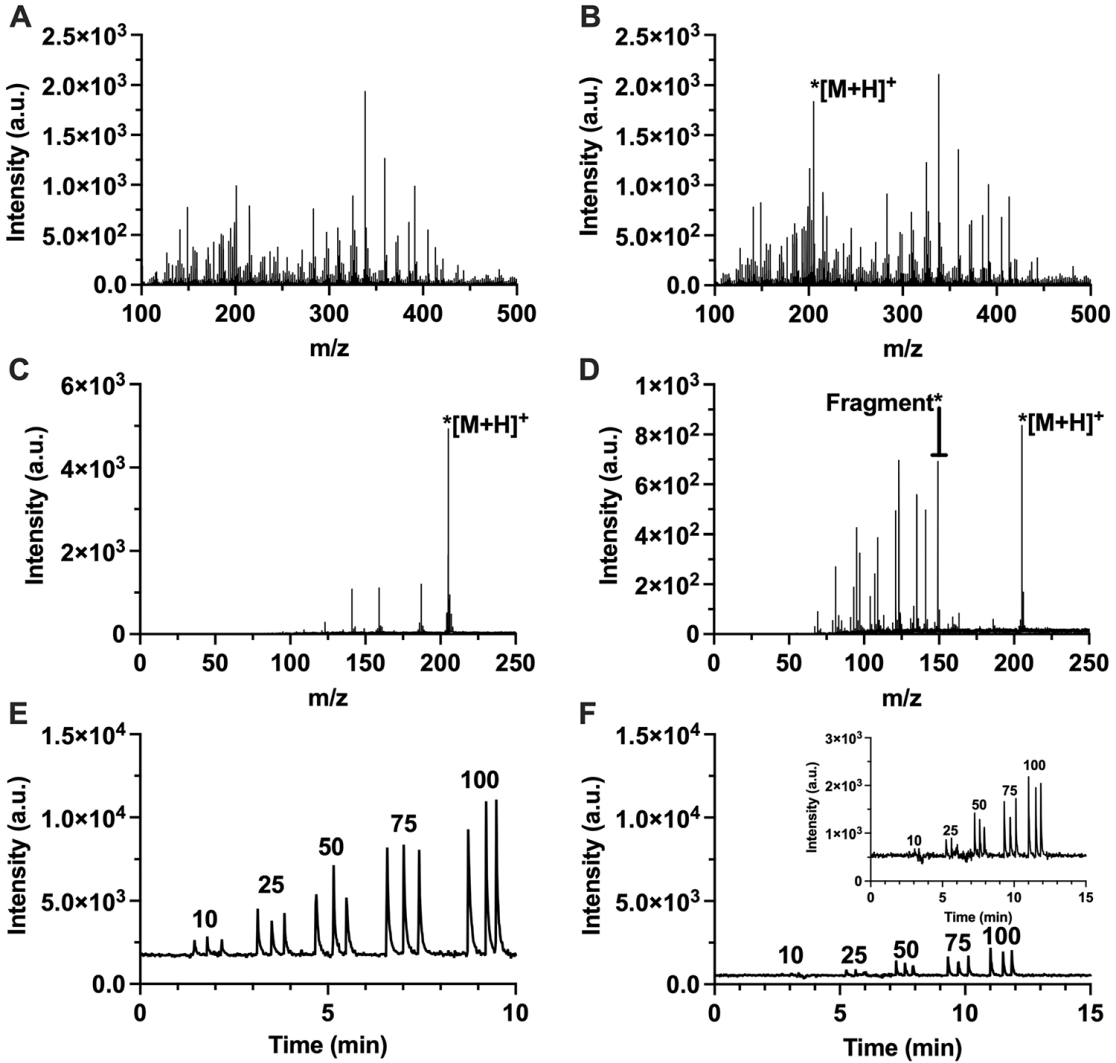
FIA-APCI-MS and FIA-APCI-MS/MS characterization of valencene with carrier phase (ethanol) infused at 1 ml min^−1^ full scan spectra where (A) no injection was performed and only carrier phase was infused and (B) where 50 μg ml^−1^ valencene was injected. (C) Full scan spectrum of 50 μg ml^−1^ valencene where quadrupole is filtering for *m/z* = 205.2 and (D) resulting fragment spectrum where collision energy = 15 V. (E) FIA-APCI-MS trace extracted at *m/z* = 205.2 of valencene calibration standards with quadrupole filtering for *m/z* = 205.2 with collision energy = 0V. (F) FIA-APCI-MS/MS trace extracted at fragment mass (*m/z* = 149.2) of same standards with collision energy = 15 V. Numbers on plot are concentration in μg ml^−1^ in panels e and f.

MS/MS was investigated to improve selectivity. Ethanol produced a complicated spectrum creating potential for interference with target analytes (Fig. 2A and B). Filtering for protonated valencene with the quadrupole led to a simpler full scan MS spectrum (Fig. 2C). Varying the collision energy revealed that 15 V produced the highest MS/MS signal for the 149.2 fragment, which was used for valencene quantiflcation (Fig. 2D). The MS method produced greater signal than MS/MS (Fig. 2E and F). Similarly, MS yielded better signal to noise ratio (S/N). For the 100 μg mL^−1^ valencene standard, S/N was 110 ± 13 for MS and 56.2 ± 4.8 for MS/MS, and 10 μg mL^−1^ valencene is clearly distinguished from the noise with MS but not MS/MS. Despite the better S/N for quadrupole-flltered MS detection, MS/MS was used for higher selectivity in further experiments; however, if sensitivity was limiting MS is likely feasible.

**Fig. 3:**
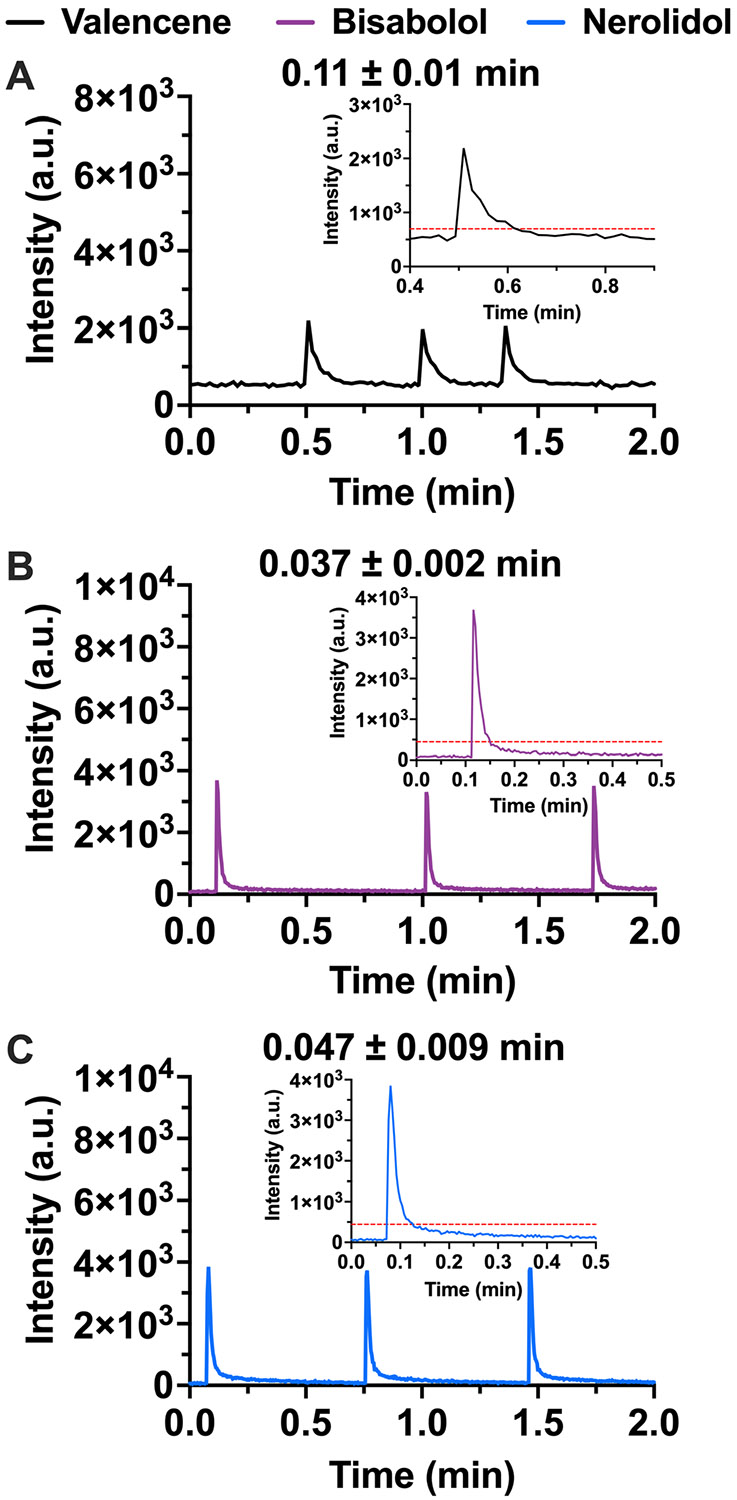
FIA-APCI-MS/MS traces for 100 μg ml^−1^ triplicate injections of (A) valencene (black trace, S/N = 109 ± 5), (B) bisabolol (purple trace, S/N = 726 ± 172), and (C) nerolidol (blue trace, S/N = 725 ± 124) standards. Average width at 10% of peak height ± standard deviation (*n* = 3) is noted in upper portion of plots. The insets show a zoomed in view of a single peak with a dashed red line plotted at 10% of the peak height.

MS/MS experiments were then conducted on bisabolol and nerolidol in the same manner as above. Using the tuned MS/MS settings, the widths obtained at 10% of the peak height for 100 μg mL^−1^ standards for valencene, bisabolol, and nerolidol were compared. Widths ranged from 2.4 to 6.6 s at 10% of height (Fig. 3). It is likely that similar throughputs (*i.e*., an injection every 20 s) could be achieved for all three terpenes if the tail of the peak can be ignored. Fermentation reactions producing bisabolol and nerolidol were then investigated to evaluate method accuracy.

The direct MS/MS method used here is fast; however, it may not be suitable if isobaric species must be resolved or if matrix effects vary from sample to sample. The present method was not required to separate isobaric species, as each strain variant investigated produced either only bisabolol or nerolidol. MS/MS may not be sufficient to distinguish between isobaric terpenes because of their similar fragmentation patterns.^42-44^ Ion mobility has been employed as a separation step prior to MS detection of isobaric terpenes,^45^ which would provide separation benefits while maintaining the method's high throughput. In a typical screening experiment, the matrix effects are likely to be consistent across samples and therefore a separation step may not be necessary; however, if the matrix varies then a separation step or more sample preparation would be required.

### 3.2 Repeatability of calibration curves and detection of bisabolol in fermentation extracts using FIA-APCI-MS/MS

**Fig. 4:**
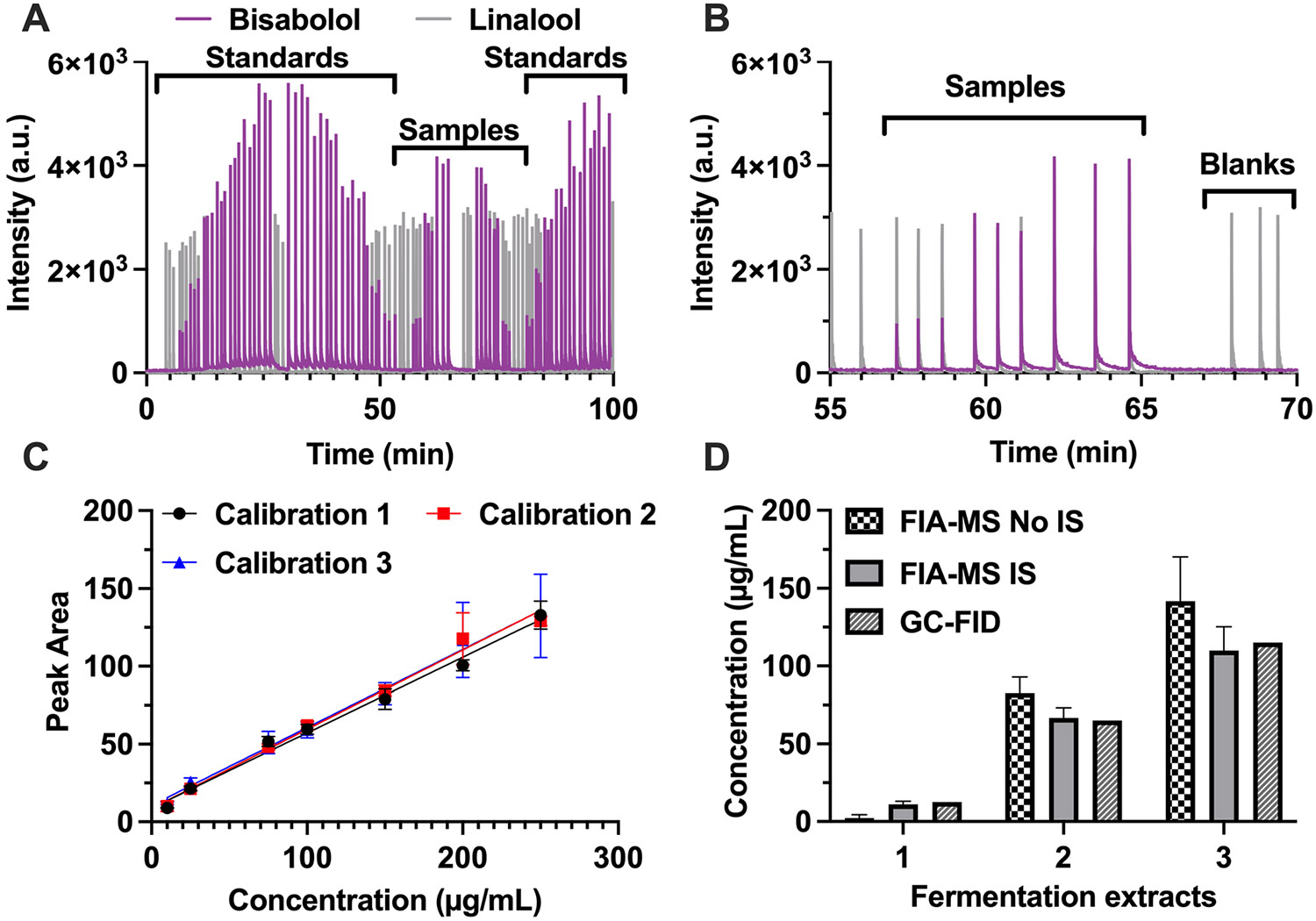
FIA-APCI-MS/MS analysis of strain variants producing bisabolol. All samples and standards were injected in triplicate. (A) Full FIA-APCI-MS/MS trace including calibration standards and 3 strain variants producing varying amounts of bisabolol (purple trace) with is linalool trace (gray) added. (B) Zoomed in view where strain variant samples and blanks were infused. (C) Calibration curves generated from analysis. The entire set of calibration standards was injected three times over the course of the run. Calibration curve 1 covers the injections from 0–30 min. Calibration curve 2 covers the injections from 30–55 min. Calibration curve 3 covers the injections from 80–100 min. Points are plotted as average ±1 SD for replicates within one calibration curve (*n* = 3). (d) Comparison of fia-apci-ms ms^−1^ with and without is to GC-FID for bisabolol concentration. For FIA-MS data, bars are plotted as average of *n* = 3 injections ±1 SD. GC-FID concentration was determined from a single injection, so no error bars are plotted.

The method was evaluated by detecting bisabolol from fermentation extracts (Fig. 4). For this example, a sequence of 97 injections injected at 1 min intervals was performed including: (1) a series of increasing concentration bisabolol standards over 10–250 μg mL^−1^ analyzed in triplicate for calibration; (2) a reverse order of the same samples to test the calibration result; (3) triplicate analysis of 3 different strain variants producing bisabolol *via* fermentation; (4) repeat of the first calibration standards; and (5) blanks that were injected in triplicate throughout the sequence (Fig. 4A). Fig. 4B shows a zoomed in view of the resulting data trace where the fermentation extracts were injected. The signals from the three standard series were plotted as separate curves revealing linear and repeatable calibration curves (Fig. 4C). The slope of the 3 calibration curves had an RSD of 2.5% indicating that the response and sensitivity remained stable over the entire sequence of injections. Using the limit of the blank method,^46^ the limit of detection is calculated to be 1.4 μg mL^−1^. This LOD is more than adequate for screening where relatively high concentrations are expected. Sufficient LOD was obtained even though dodecane, which was present in all standards and samples at 1.25%, has been found to suppress ionization in APCI.^14^

The data from the injection sequence also show that the method gave good agreement with GC-FID analysis for the 3 strain variants that were analyzed, as illustrated in Fig. 4D. The results show that the IS improved agreement between the methods (Fig. 4D). For instance, all three samples were within 11% of the concentration determined by GC-FID when the IS was used. When the IS was not used, samples were up to 82% different from the GC-FID concentration. The normalization particularly helped for the lowest concentration sample, with this sample being the most different from GC-FID in terms of concentration and having the highest RSD. Using the normalization, the accuracy (determined as percent difference from the GC-FID method) was 11%, 3%, and 4% and the precision (determined as percent RSD) was 18%, 10%, and 14% (*n* = 6) for the three samples represented in Fig. 4D.

Blank samples, containing the IS, were infused at the beginning and end of the run, as well as after the first two calibration curves. Analyte carryover was not observed in any of these injections. These results show that FIA-APCI-MS/MS is a useful method for analyzing terpenes in fermentation extract at a throughput of 1 injection/min, faster than standard screening techniques.

### 3.3 Screen of 99 strain variants for nerolidol using FIA-APCI-MS/MS

**Fig. 5:**
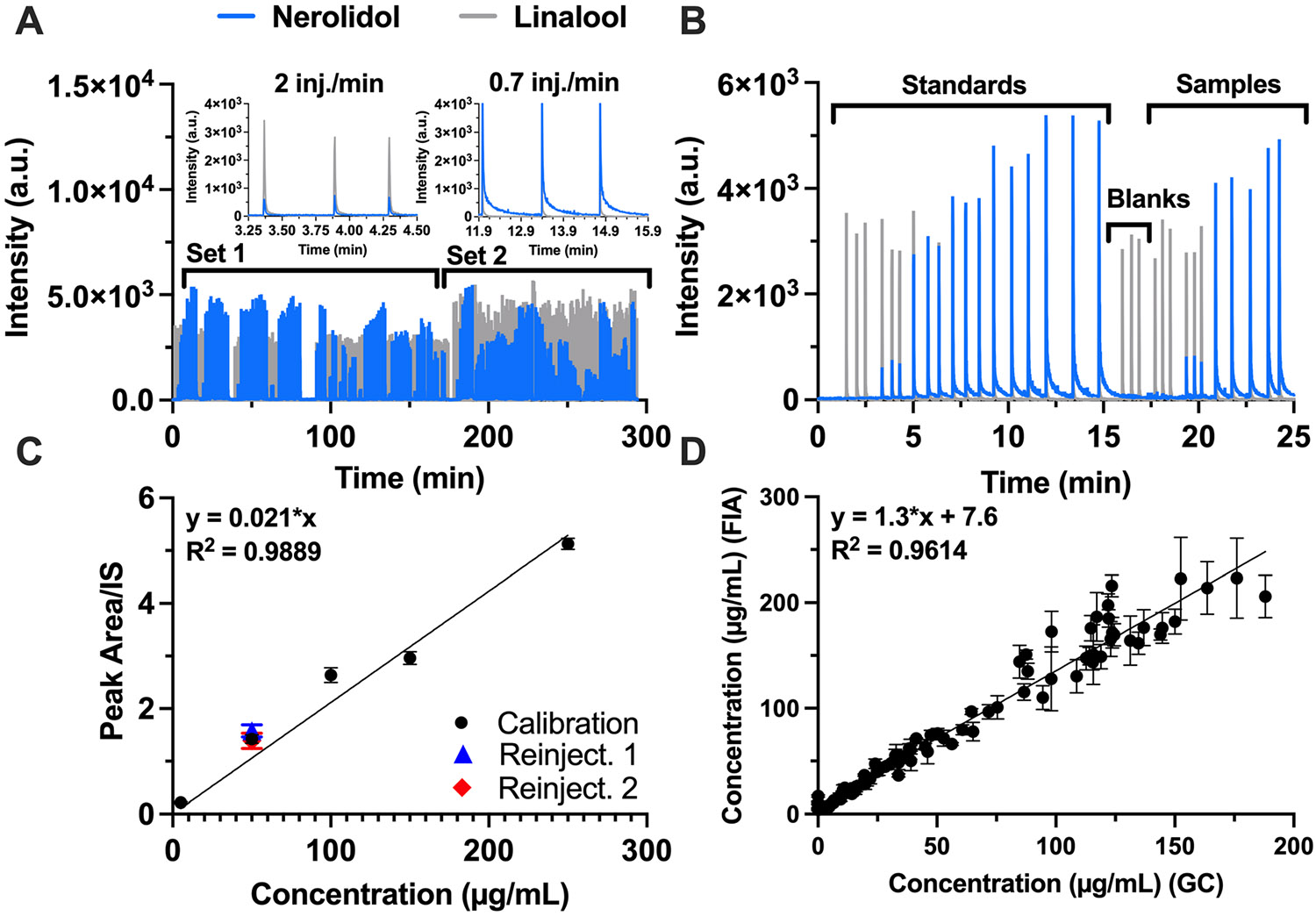
(A) Full FIA-APCI-MS/MS trace including calibration standards and 99 strain variants producing varying amounts of nerolidol (blue trace) with is trace shown (gray). Set 1 represents data from one experimental run (51 reactions) and set 2 represents data from another (48 reactions). Insets show where throughputs of 2 injections per min and 0.7 injections per min were achieved. (B) Zoomed in view where calibration standards and first 4 strain variant samples are infused. (C) Plot of calibration curve from set 1. Points are plotted as average ±1 SD (*n* = 3). A linear regression was performed on the data and fitted through the origin, yielding the equation *y* = 0.021 × *x* and *r*_2_ = 0.9889. In legend, calibration refers to initial calibration, reinject. (1) Refers to the first reinjection of the 50 μg ml^−1^ standard, and reinject. (2) Refers to the second reinjection of the 50 μg ml^−1^ standard within set 1. (D) Correlation between FIA-APCI-MS/MS and GC-FID for nerolidol concentration over 99 samples. FIA concentrations are plotted as average ± SD of *n* = 3 injections. A regression was performed on the data yielding the equation *y* = 1.3 *x* + 7.6 and *r*_2_ = 0.9614.

To evaluate the method for screening a larger number of different samples, nerolidol content was determined in 99 fermentation extracts, where each reaction mixture contained a different strain variant, analyzed in triplicate. Dodecane extracts from fermentation mixtures were undiluted if concentration was inside the linear range of the method or diluted 300-fold in dodecane if not. (Concentrations of the fermentation extracts were known prior to FIA-APCI-MS/MS analysis; however, a related dilution could be performed if the yield of the un-engineered strain is known and the only interest is to find variants with improved yield). A trace from the sequence of injections covering the 99 samples, which includes two sets of calibration standards over 5–250 μg mL^−1^, is shown in Fig. 5A. Fig. 5B shows one set of calibration standards acquired within the initial injections, and the resulting calibration curve is shown in Fig. 5C. A 50 μg mL^−1^ standard was reanalyzed at the middle and end of analysis in set 1 to assess stability of the method (Fig. 5A). The IS normalized peak areas were within 11% of the initial normalized peak area suggesting reasonable stability. No carryover was detected in any of the blanks across the two sample sets that were infused after the calibration curve, during the middle of sample analysis, or at the end of sample analysis as shown in Fig. 5B.

Concentrations of nerolidol from the FIA-APCI-MS/MS screen were compared to those obtained *via* GC-FID over a total of 99 strain variants (Fig. 5). Good agreement (*R*^2^ = 0.96) was observed between the two methods over 99 samples run in triplicate as seen in Fig. 5D, indicating that the FIA-APCI-MS/MS method can perform accurate relative quantification when compared to the GC-FID method, which required quadruple the analysis time (4.2 min) with an injection to injection time of 7–8 min due to time needed for thermal re-equilibration of the instrument. The FIA-APCI-MS/MS method also provided consistent response over triplicate injections (average RSD = 11%). The method cannot perform absolute quantification, as the average percent difference between concentration values determined by FIA-APCI-MS/MS and GC-FID was 50%. However, relative quantification is sufficient for screening experiments.^12^ Sample preparation for the two methods is the same, but analysis time for 195 injections is 173 minutes (experimentally determined time observed in Fig. 5A) for FIA-APCI-MS/MS and 1281 minutes (based on 7 min per sample throughput) for GC-FID.

The throughput of this method was 1.3 injections per min, which is calculated by dividing the total number of injections for all 99 strain variants (*n* = 375 injections, which is 99 samples, 26 calibration standards, and blanks all analyzed in triplicate) divided by the time for the total injection sequence. Analysis could be further increased without sacrificing accuracy by performing single injections instead of triplicate (Fig. S6), which would allow for a 3-fold reduction in analysis time. Timing of the injections was varied according to the signal observed *i.e.*, as signal returned to baseline the next injection was initiated. As shown in the insets of Fig. 5A, higher concentration samples generate wider peaks than lower concentrations resulting in longer times before the next injection. The lowest concentration standard, 5 μg mL^−1^, was injected at 2 injections per min whereas the highest concentration, 250 μg mL^−1^, was injected at 0.7 injections per min (Fig. 5A). In the screens shown, a conservative approach was used in that injections were timed to enable peak area measurement without interference from subsequent injections for the best quantification.

Throughput could be increased by diluting samples. If the yield of the starting strain is known, then it would be possible to dilute samples such that product concentration of the starting strain is at the lower end of the linear range of detection. Such dilution would allow the majority of strain variants to be within this low concentration region, since most variants do not outperform the starting strain if random mutagenesis strategies, like directed evolution, are used.^47^ As a result, the average width would be smaller and allow faster injections *e.g.*, up to 2 injections per min. Given that the FIA-APCI-MS/MS and GC-FID data appears to be well correlated at lower concentrations, this approach would not sacrifice relative quantification accuracy.

If the motivation is to simply identify the highest producing strains as opposed to quantifying terpene content in all samples, then it would also be possible to overlap the injections i.e., inject while the tail ofa previous injection is being detected. The FIA-APCI-MS/MS results plotted in Fig. 5D were obtained by integrating the peak area within the first 20 s of the peak even if the peak tail was still being detected after 20 s, indicating that the tail of the peak is not needed for accurate relative quantification as evidenced by the high correlation (*R*^2^ = 0.96) observed in Fig. 5D. As shown in Fig. S7, the tail area after 20 s represents less than 1% of the total peak area for the highest concentration detected. If peaks are overlapped to increase throughput, it is also important to consider whether the tail area of the sample affects the relative quantification of the next sample.^48^ To evaluate this, the peak tail area was quantified in the peaks associated with the strain variant producing the highest concentration of nerolidol, as these peak tails should be the largest, and compared to the peak area of the strain variant with the smallest concentration of nerolidol, as the peak tail effect should be the largest for this sample (Fig. S7). In this worst-case scenario, the calculated nerolidol concentration for the lower concentration sample (*i.e.*, sample affected by the tail of the higher preceding sample) would only increase by 5%. This demonstrates that the potential increase in area caused by injecting samples every 20 s would not significantly impact accuracy of the method for screening.

### 3.4 Discussion on FIA-MS/MS throughput and potential for improvement

The throughput of this method is comparable to the fastest previous methods reported for similar compounds (*e.g*., 1 min per sample for PTR-MS of valencene samples *via* headspace injection,^14^ 1 min per sample for ergosterol with direct liquid injection APCI-MS/MS^35^) (Table 2); however, the present method notably differs from these existing methods because it offers similar throughput from four times less sample volume and has potential to be improved over 2-fold. This method was also the first demonstrated to be stable for a larger number of samples *(n* = 99 samples in present study, whereas *n* = 4 in previous studies^14,35^). With specific regard to the PTR-MS method, the present method allows for MS/MS analysis from a liquid sample. MS/MS analysis is attractive in cases where a complicated background is present, as in the current study (Fig. 2A and B), to increase measurement confidence. Furthermore, the present method allows for analysis of liquid sample, which can be helpful for terpene analysis where loss of analyte has been observed with headspace techniques.^49^ In this study, throughput was limited by the peak width, which was also reported to be the case for PTR-MS analysis of valencene samples.^14^ As discussed above, it is feasible to increase throughput up to 2.3-fold with little modification of the method.

## 4. Conclusions

In this work, we introduce a rapid method for analyzing terpene content in fermentation extracts. Using FIA-APCI-MS/MS, it was possible to detect bisabolol and nerolidol in fermentation extracts at a throughput of ~1 injection/min with no detectable carryover. A screen of 99 extracts containing nerolidol produced via fermentation revealed that the correlation between the FIA-APCI-MS/MS and GC-FID methods in terms of concentration was good (*R*^2^ = 0.96). Strategies to further increase throughput of the method include: diluting samples such that most fermentation extracts contain analyte within the lower region of linear detection and performing injections every 20 s such that peak tails overlap. We envision this technology as being useful for relative quantification in screens aimed at engineering microorganisms or enzymes to produce nonpolar compounds, as it is an improvement in terms of throughput upon traditional screening techniques (*e.g*., GC-MS).

## Supplementary Material

supplementary material

Supplementary information (SI): analyte structures, effect of carrier phase on peak width, effect of injection volume on peak width, effect of concentration on peak width, correlation between FIA and GC methods for single injections, and peak tail area figure. See DOI: https://doi.org/10.1039/d5ay02027a.

## Figures and Tables

**Table 1:** MS parameters for terpene analysis

	Quadrupole set mass (*m/z*)	Collision energy (V)	Scan time (s)^a^	Mass range (*m/z*)	Fragment (*m/z*)^b^
Valencene	205.2	15	1	20–500	149.2
Bisabolol	205.2	15	0.1	20–500	121.1
Linalool	137.2	10	0.1	20–500	81.1
Nerolidol	205.2	15	0.1	20–500	121.1

a Scan time is duration over which TOF ion detections accumulate in each individual spectrum presented to MassLynx.

b The fragments 149, 81, and 121 have been detected previously as fragments of terpenoids.^16,50,51^

**Table 2:** Comparison of GC-FID, PTR-MS,^14^ and FIA-APCI-MS/MS methods for valencene analysis

	GC-FID	PTR-MS^14^	FIA-APCI-MS/MS
Limit of detection	79 μg mL^−1a^	Not reported	2.3 α 0.6 μg mL^−1^ (*n* = 2)
Flow rate	40 mL min^−1^	52 mL min^−1^	1 mL min^−1^
Carrier phase	Gas	Gas	Liquid
Throughput	0.1 injections per min	~1.0 injections per min	1.8 injections per min
Volume of sample required	1 μL^b^	200 μL^c^	1 μL^d^

a Typically value but can be lowered by varying split ratio.

b Typically 60 μL is loaded into the vial to perform a 1 μL injection.

c Headspace injections were performed off 200 μL of sample in the case where 96 well plates were used.

d Sample loop was overfilled with 5 μL to ensure repeatable injections, but only 1 μL was injected.

## Data Availability

Full data sets are available upon reasonable request to the corresponding author
