# Supplementary material for: Rapid analysis of terpenes produced by fermentation using flow injection analysis coupled to APCI MS
Source: Anal Methods. Author manuscript; Available in PMC 2026 Mar 5. (PMC12961749; doi:10.1039/d5ay02027a)
Supplement: supplementary material [file NIHMS2145327-supplement-supplementary_material.pdf]

Supplementary Information (SI) for Analytical Methods.
This journal is © The Royal Society of Chemistry 2026

Supplementary Information

**Rapid analysis of terpenes produced by fermentation using flow injection analysis coupled to APCI MS**

Bridget E. Murray[1], Moritz Pott[2], Jules Beekwilder[3], and Robert T. Kennedy[1,4,*]

[1]*Department of Chemistry, University of Michigan, Ann Arbor, MI 48109, United States*
[2]*BASF SE, 67056 Ludwigshafen am Rhein, Germany*
[3]*Isobionics BV, 6167 RD, Geleen, The Netherlands*
[4]*Department of Pharmacology, University of Michigan, Ann Arbor, MI 48109, United States*

*Corresponding Author.
*Email Address*: rtkenn@umich.edu

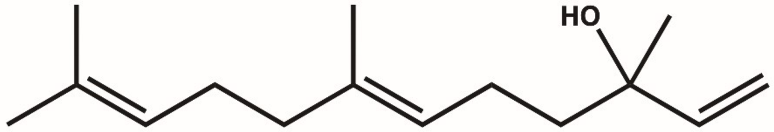


**Nerolidol**

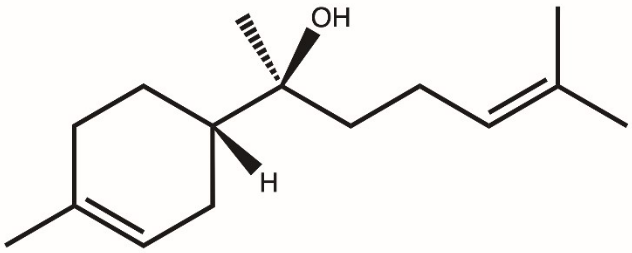


**Bisabolol**

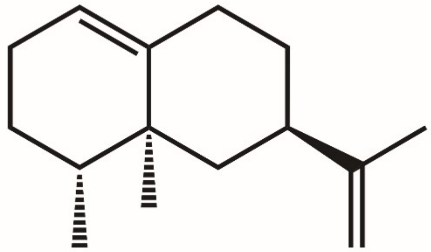

**Valencene**

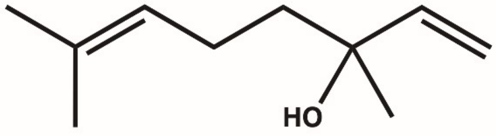


**Linalool**

**Fig. S1**. Analyte structures.

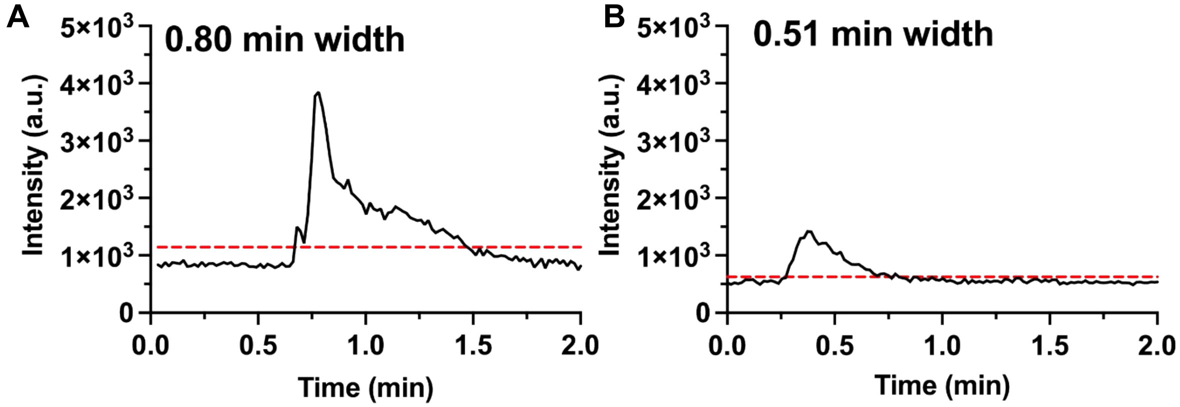


**Fig. S2**. Effect of (A) water and (B) ethanol as carrier phase on FIA-APCI-MS peak widths for 1 μL injection of 10 μg/mL valencene in ethanol infused at 0.05 mL/min. Value in upper portion of panels is width at 10% of peak height (n = 1), indicated by the dashed red line. Traces were extracted at m/z = 205.2. MS settings were: corona current =10 μA, sampling cone = 33, extraction cone = 2.0, source temperature = 100 ˚C, probe temperature = 400 ˚C, cone gas flow = 0 L/h, desolvation gas flow = 600 L/h.

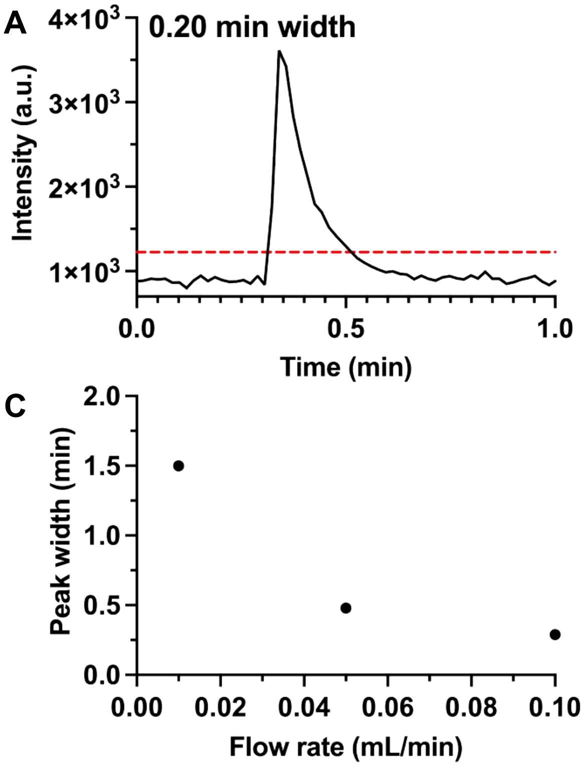


**Fig. S3**.Effect of (A) 0.5 mL/min and (B) 1 mL/min carrier phase flow rate on FIA-APCI-MS peak widths for 1 µL injection of 50 µg/mL valencene. Width at 10% of peak height is noted in upper portion of panels (n = 1) and indicated by dashed red line. (C) Plot of peak width vs. flow rate for 0.01, 0.05, and 0.10 mL/min flow rates for 10 µg/mL standard (n = 1). Traces were extracted at m/z = 205.2. MS settings were: corona current = 10 µA, sampling cone = 33, extraction cone = 2.0, source temperature = 100 ˚C, probe temperature = 400 ˚C, cone gas flow = 0 L/h, desolvation gas flow = 1200 L/h.

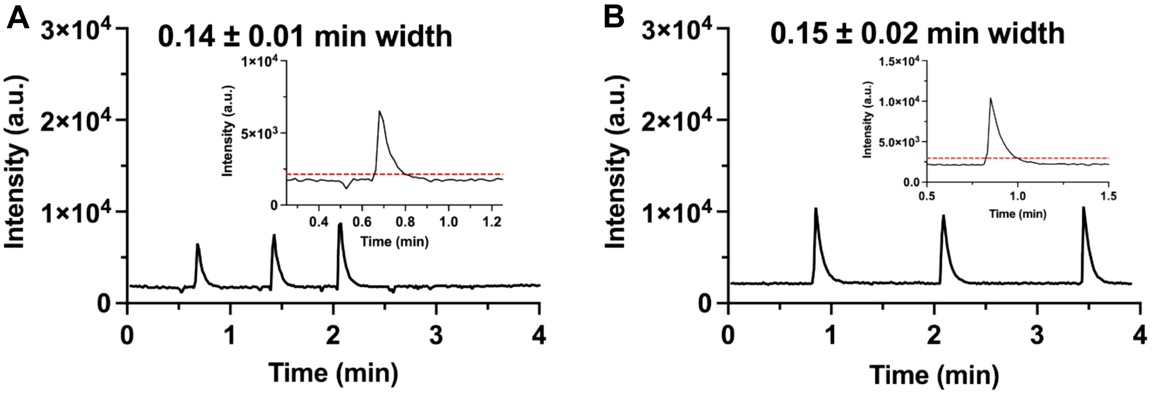


**Fig. S4**. Effect of (A) 0.3 µL sample loop and (B) 1 µL sample loop on FIA-APCI-MS peak width for 50 µg/mL valencene standard infused at 1 mL/min. Traces were extracted at m/z = 205.2. Average width at 10% of peak height ± standard deviation (n = 3) is noted in upper portion of panels. The insets show a zoomed in view of a single peak with a dashed red line plotted at 10% of the peak height.

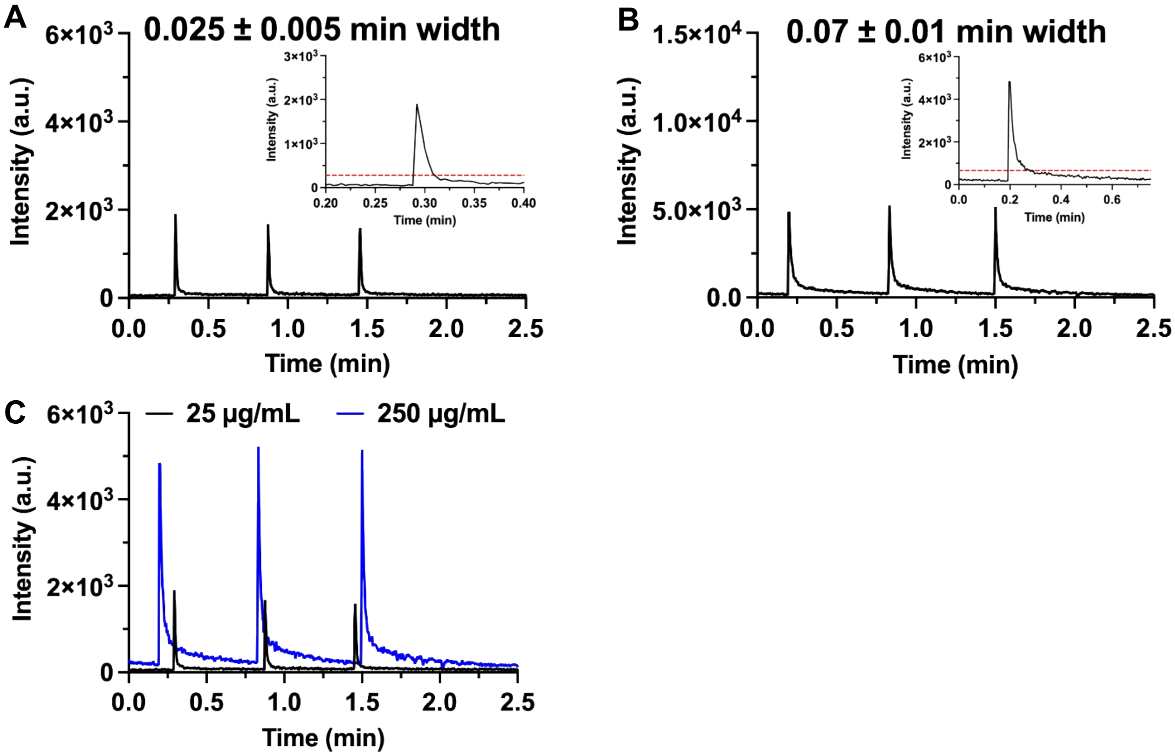


**Fig. S5**. Triplicate injections of (A) 25 µg/mL and (B) 250 µg/mL bisabolol using FIA-APCI-MS/MS. Average width at 10% of peak height ± standard deviation (n=3) is noted in upper portion of panels. The insets show a zoomed in view of a single peak with a dashed red line plotted at 10% of the peak height. (C) Overlay of traces in (A) and (B), where black trace represents 25 µg/mL and blue trace represents 250 µg/mL. Quadrupole was set to m/z = 205.2 and traces were extracted at m/z = 121.1.

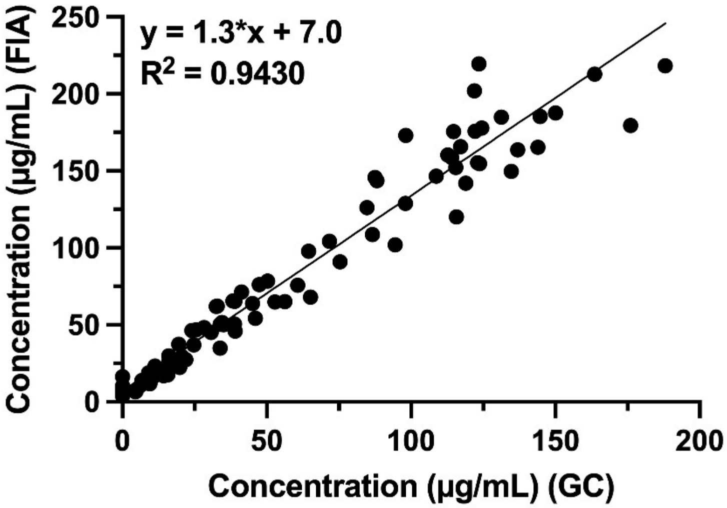


**Fig. S6**. Correlation between FIA-APCI-MS/MS and GC-FID for nerolidol concentration over 99 samples using single injection data for FIA concentrations. A regression was performed on the data yielding the equation y = 1.3 * x + 7.0 and $R^2$ = 0.9430.

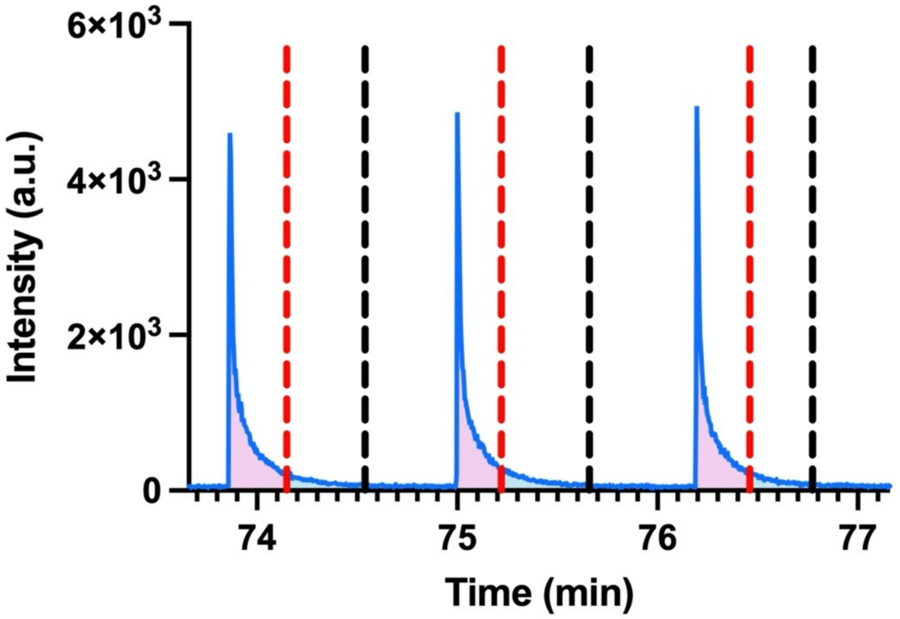


**Fig. S7**. FIA-APCI-MS/MS trace showing triplicate injections of the strain variant extract containing the highest concentration of nerolidol (blue trace). The pink shaded region, demarcated by the red dashed line, shows the peak area that was used for quantification of nerolidol. The blue shaded area, demarcated by the black dashed line, shows the additional peak tail area.
